# Spectator Motives and Points of Attachment: an Investigation on Professional Basketball

**DOI:** 10.2478/v10078-011-0087-9

**Published:** 2011-12-25

**Authors:** R. Timucin Gencer, Olcay Kiremitci, Hayal Boyacioglu

**Affiliations:** 1 Ege University, School of Physical Education and Sports, Department of Sport Management; 2 Ege University, School of Physical Education and Sports, Physical Education and Sports Teaching Department; 3 Ege University, Faculty of Science, Department of Statistics

**Keywords:** basketball, spectator attendance, effectiveness, strategy

## Abstract

Spectator attendance to professional basketball in Turkey is significantly less than desired. Keeping in mind how important spectators are for team sports, understanding factors that affect game attendance will offer essential clues in terms of increasing spectator attendance. The main purpose of this study was to determine the relationships between basketball spectators’ motives and points of attachment. With consideration to this purpose, the present study has tested the validity and reliability of the Motivation Scale for Sport Consumption and the Points of Attachment Index for Turkish basketball spectators. 197 basketball spectators participated in the study. Confirmatory factor analysis results demonstrated that the original models of the measurement tools employed for the study showed an acceptable degree of fit with the data. The internal consistency coefficients of the scales were found to be between 0.59 and 0.80 for the Motivation Scale for Sport Consumption and between 0.53 and 0.88 for the Points of Attachment Index. The canonical correlation analysis only returned a single significant function. The motives aesthetics and escape stood out in terms of the significant function, while the sport type (basketball in this study) stood out in the sense of attachment. Relationships identified between basketball spectators’ motives and points of attachment could help sports managers and marketing experts to develop strategies focusing on increasing spectator attendance to their teams’ games.

## Introduction

The social development process in the post-industrialisation era has contributed significantly to the development of sports (Amman, 2000). In particular, a rise in prosperity has promoted the development of a wide range of options for individuals to spend their leisure time. The 20^th^ century encountered modern sports transforming into the activity of the masses, now making up a significant share of the wide range of options developed for individuals to spend their spare time (Gouguet, 2006). In context of individuals’ choice for spending leisure time, the popularity of spectator-based professional sports disciplines is getting more and more important.

In the context of professional sports organisations, spectators play an indispensible role not only in the sense of direct and indirect income (Mullin et al., 2000), but also in increasing performance on the field (Courneya & Carron, 1992; Polard & Polard, 2003). Therefore it becomes very important to identify factors that influence individuals’ attendance as spectators to the event on offer (Zhang et al., 1995; Trenberth & Garland, 2007; Cunningham & Kwon, 2008).

In financial terms, spectators’ attendance to sports games is influenced by a number of factors including ticket price, level of income, cost of alternative products, market size, and the significance and unpredictability of the end result (Simmons, 2006). However, sports are about an experience, regardless of how consumer behaviour is evaluated. The pursuit for consuming this experience reflects a desire to satisfy personal needs and gain certain benefits (Funk, 2008). Therefore individuals may wish to attend a sports game as a spectator for very different reasons. This is closely related to the individual’s psychological and social needs, which is why a large number of studies conducted on spectator motives focused on those needs (Trail et al., 2000; Zhang et al., 2001). Motives that satisfy such needs include vicarious achievement, aesthetics, drama, escape, acquisition of knowledge, physical skills of the athletes, and social interaction (Trail & James, 2001).

Identification has been defined as “an orientation of the self in regard to other objects, that results in feeling or sentiments of close attachment” (Trail et al., 2000). Various authors have reported the significance of identification in terms of sports consumption behaviour (Wann & Branscombe, 1993; Trail et al., 2000; Matsuoka et al., 2003). Most of these studies investigated spectators’ level of team identification. However, identification to a certain team may not be enough to ensure a spectator’s attendance to the game. A number of attachment points such as team, players, coach, society, type, and level of sports could also be of great importance for the spectator (Robinson & Trail, 2005).

After professional football, professional basketball is the second most popular type of sports in Turkey. Turkey’s biggest team sports achievements at the international level have taken place in basketball. An increase in income from both sponsorship and broadcast rights over the past decade has supported club efforts in assembling much better teams. Regardless of all these developments, spectator attendance to the Beko Turkish Basketball League is significantly less than desired. Having competed in the major league since the establishment of the Turkish Basketball League, the Karşıyaka Sports Club basketball team is one of the clubs that benefits most from spectators’ direct and indirect contribution. Therefore, having an understanding about the basketball game-related motives and attachment points of this specific spectator group will offer guidance to developing marketing strategies focusing on increasing spectator attendance for other teams.

Besides understanding the motive behind a spectator spending time and money on a specific sports game, there is also a need to clarify the main factor engaging the spectator to the event itself (Robinson et al., 2004). This study has two objectives: first, to test the validity and reliability of the largely popular Motivation Scale for Sport Consumption (Trail & James, 2001) and Points of Attachment Index (Trail et al., 2003) on Turkish basketball spectators; second, to test the relationships between sports consumption-related motives and points of attachment.

## Material and Methods

In total 197 professional basketball game spectators, of which 155 were male and forty-two were female, participated in the study. The mean age of the participants was 25.36±8.84 years. The study employed two different measurement tools – the Motivation Scale for Sport Consumption (Trail & James, 2001) and the Points of Attachment Index (Trail et al., 2003). The Motivation Scale for Sport Consumption (MSSC) consists of twenty-one items with seven sub-dimensions (vicarious achievement, aesthetics, drama, escape, acquisition of knowledge, physical skills of the athletes, and social interaction). The second measurement tool, the Points of Attachment Index (PAI) consists of twenty-three items and seven sub-dimensions (players, team, the coach, society, sports discipline, city, and level of sports competition). Both tools are based on a seven-point Likert scale.

In order to determine whether the items functioned adequately in line with reliability criteria, an item analysis based on the mean difference of the upper and lower 27% groups was performed initially as part of the adaptation process. This was followed by internal consistency coefficients calculations. Confirmatory factor analysis (CFA) was used to test the structural validity of both measurement tools. The canonical correlation analysis was performed with the purpose of demonstrating the relationships between the sports consumption related motives and the points of attachment variable sets.

## Results

The item analysis concluded that the t values of items constituting both measurement tools were statistically significant (p<0.05), meaning that all items were functional (Table 1–2).

Considering internal consistency coefficients, the Cronbach’s alpha coefficients were between 0.59 and 0.80 for the MSSC and between 0.53 and 0.88 for the PAI. Examining the sub-dimensions that constitute the MSSC, the motives vicarious achievement, drama, aesthetics, and physical skills respectively have the highest mean values. For the PAI; the team, sports discipline, society, and the level of sports discipline respectively have the highest mean values in terms of points of attachment (Table 3).

Once both scales were identified to comply for reliability, their structural validity was tested with CFA. Fit indexes determined by measurement results were RMSEA= 0.067, SRMR= 0.084, χ^2^=386.58, df=205, χ^2^/df= 1.88 for the Motivation Scale for Sport Consumption and RMSEA= 0.073, SRMR= 0.086, χ^2^ =335.25, df=163, χ^2^/df =2.05 for the Points of Attachment Index. On the other hand, R^2^ values varied between 0.11 and 0.75 for MSSC and between 0.17 and 0.82 for PAI (Table 4).

The canonical correlation analysis was performed to demonstrate the relationships between the variable sets sports consumption related “motives” and “points of attachment”. The first of the calculated canonical correlation coefficients (0.57) was found to be statistically significant (p<0.05) (Table 5).

In regard to the canonical loadings of points of attachment related variables, the greatest contribution came from the “type of sports” variable (0.809). Examining the canonical cross loadings of the attachment related variable, again the greatest contribution came from the type of sports variable (0.461).

In the canonical loadings of motives related variables, the greatest contribution came from the escape variable (0.809). Among the canonical cross loadings of the attachment-related variables, again the greatest contribution came from the variables “escape” (0.461) and “aesthetics” (0.444) (Table 6).

A redundancy analysis was employed for two purposes: first, to investigate the extent canonical variables that are points of attachment and motives which are able to explain total variation; second, to understand how much the variation of each can be explained by the other variable. The results showed that the rate of explained variance for the attachment variable was 0.267 with a redundancy index of 0.087 and the rate of explained variance for the motives variable was 0.387 with a redundancy index of 0.126 (Table 7).

## Discussion

The current study differs from previous studies (James & Ridinger, 2002; Trail et al., 2003), as spectators making up the sample group are those attending professional basketball games. The majority of studies on spectator consumption in the United States are conducted in leagues within the NCAA organization. In terms of organization these leagues, in many ways, show similarities to professional leagues in Europe. Obtaining qualitative data on spectator consumption in Turkey and in many European countries is only possible with professional sports league spectators. Although this fact implies certain difficulties in the data collection process, it is very important in terms of the efficacy of the spectator-oriented strategies which professional sports teams develop.

The CFA analysis was employed to investigate the structural fit of measurement tools to be utilised in the first part of the study on the sample used for scaling. In scope of the applied CFA criteria, Chau (1997) reported that the rate of chi-square to degree of freedom being equal or smaller than 3 is adequate for good fit. On the other hand, Schermelleh-Engel et al. (2003) argued that rates between 2 and 3 should be considered as an acceptable value. Considering fit indexes, Hu and Bentler (1999) pointed out that RMSEA values between 0.06 and 0.08 indicate an acceptable degree of fit. Schermelleh-Engel et al. (2003) suggested that SRMR values smaller than 0.10 could be interpreted as an acceptable value. In respect to other fit indexes CFI values between 0.90 and 0.95 (Hu & Bentler, 1999), and GFI values between 0.85 and 0.95 (Marsh et al., 1988) are considered an indication of acceptable fit.

Besides discovering a significant t-value (p<0.05) from the item analysis results of the study scales, finding a >0.70 value obtained from the internal consistency figures of the scales demonstrated that both measurement tools are structurally valid on professional basketball spectators and that they are reliable measurement tools in terms of internal consistency. However, considering the internal consistency coefficients related to the sub-dimensions of the scales, it was observed that drama (0.59) and physical skills (0.61) in MSSC, and city (0.53) in PAI returned low reliability coefficients. Tuckman (1999) reported that a Cronbach’s alpha value equal to or greater than 0.50 is acceptable since finding low internal consistency coefficients in preference based scales lacking clear true or false options can be expected. Nunnally and Bernstein (1994) reported that scales with a small number of items reduce the Cronbach’s Alpha value. In conclusion, there appears to be a need for an increase in the reliability coefficients related to the sub-dimensions of scales used in this study to develop and add new variables to these dimensions.

Examining the descriptive statistics related to the scales’ sub-dimensions, the spectators forming the sample group were fans dedicated to their team and who attended the game with the motive of vicarious achievement. The achievement motive is rooted in the need for individual achievement. Therefore, vicarious achievement is the individual taking credit for others’ achievements. Fans may feel more powerful, important and successful by means of the psychological attachment they build towards their team (Smith, 2008). It is important for sports consumers to experience feelings like self-fulfilment, self-esteem and prestige (Schwarz & Hunter, 2008). Fans feel that they deserve to experience these feelings in return for the time and money they spent at sports games. This is why spectators usually become fans of successful athletes or teams. Fans with a high degree of team identification who attend games with the motive of vicarious achievement can generate significant pressure on sports managers. This is because such fans care about the results of their team. Nonetheless, managers have little control over the team performance during the game. Henceforth, fluctuations in the team’s performance can result in important conflicts between the management and spectators with a high motivation for vicarious achievement.

One of the functions calculated by the canonical correlation analysis performed to determine the relation between data sets was found to be statistically significant. This result confirms that both measurement tools explain each other adequately and that a significant relationship exists between them. In relation to the measurement tools used in the study, this finding supports those of Robinson and Trail (2005). The investigation of canonical and cross loadings of variable pairs on spectators attending professional basketball games reveals aesthetics and escape as the dominant motive with basketball as the prevailing point of attachment. Motivation for aesthetics prompts individuals who enjoy the agility and artistic grace of athletic movements to attend games. On the other hand, the motive for escape helps individuals dissatisfied with their life at home, office, and so on to temporarily forget these issues as they consume sports under the institution of fandom. Similarly, this also prompts attendance to games (Wann et al., 2008).

Fans with a high degree of team identification may seek to experience the feeling of vicarious achievement, somewhat escaping the routine of daily life, as they watch the artistic qualities of the game in social interaction with other fans (Trail et al., 2003). The pursuit for aesthetics plays an important role in spectators’ motive for escape and game attendance. Yoshida and James (2011) reported that aesthetics has a significant effect on spectators’ service quality perception at sports events.

It could be said that spectators participating in the current study were individuals impressed by the aesthetic elements of professional-level basketball and had a desire to experience something different as they distanced themselves from the routine chores of everyday life. The obtained result is entirely based on the characteristics of the sample group and could be used in the context of marketing strategies developed for spectators with high degree of team identification. In order to develop strategies based on the relationships between spectator motives and points of attachment and be able to make generalisations, there is a need to increase the quantity of teams and spectators under investigation.

## Figures and Tables

**Table 1:** Item Analysis Results for MSSC

	**Lower %27**		**Upper %27**		**t**
	
**Items**	**mean**	**sd**	**mean**	**sd**	
**1**	1.41	1.15	4.60	2.45	−8.54*
**2**	3.41	2.00	6.26	1.36	−8.56*
**3**	2.58	1.53	4.60	1.83	−6.14*
**4**	2.90	2.22	5.66	1.70	−7.15*
**5**	4.03	1.97	6.05	1.33	−6.17*
**6**	2.18	2.05	5.41	1.95	−8.27*
**7**	3.09	2.22	6.18	1.09	−9.07*
**8**	1.45	1.20	3.94	2.34	−6.89*
**9**	5.45	2.44	6.20	1.23	−2.00*
**10**	1.15	.63	4.11	1.94	−10.53*
**11**	2.16	1.90	5.20	2.17	−7.63*
**12**	3.67	2.07	6.01	1.51	−6.63*
**13**	1.39	1.26	4.01	2.38	−7.08*
**14**	2.00	1.76	5.33	1.97	−9.19*
**15**	1.05	.23	4.00	2.19	−9.71*
**16**	4.05	2.26	6.15	1.24	−5.89*
**17**	1.24	1.15	3.67	2.11	−7.36*
**18**	2.56	2.16	5.98	1.40	−9.63*
**19**	3.32	2.35	6.26	1.19	−8.12*
**20**	3.18	2.11	5.64	1.58	−6.76*
**21**	2.64	2.32	6.07	1.57	−8.90*

* p<.05

**Table 2:** Item Analysis Results for PAI

	**Lower %27**		**Upper %27**		**t**
	
**Items**	**mean**	**sd**	**mean**	**sd**	
**1**	5.03	2.13	6.69	1.04	−5.09*
**2**	5.11	1.92	6.79	.59	−6.05*
**3**	6.13	1.49	6.86	.58	−3.33*
**4**	4.07	1.84	6.69	1.01	−9.06*
**5**	4.24	1.96	6.64	1.11	−7.74*
**6**	4.81	1.98	6.86	.52	−7.30*
**7**	3.81	2.11	6.32	1.34	−7.30*
**8**	5.37	2.22	6.88	.50	−4.82*
**9**	5.03	1.89	6.84	.41	−6.81*
**10**	5.86	1.54	6.75	.97	−3.53*
**11**	4.41	1.87	6.90	.35	−9.50*
**12**	4.05	2.12	6.73	.71	−8.70*
**13**	5.15	1.98	6.73	.71	−5.47*
**14**	4.35	2.08	6.39	1.21	−6.14*
**15**	6.07	1.67	7.00	.00	−4.02*
**16**	5.64	1.52	6.84	.45	−5.53*
**17**	5.11	1.66	6.20	1.57	−3.48*
**18**	4.71	1.76	6.83	.50	−8.35*
**19**	4.16	1.86	6.83	.57	−9.90*
**20**	5.58	1.56	6.96	.27	−6.32*
**21**	4.22	2.12	6.47	1.15	−6.75*
**22**	5.37	1.88	6.96	.19	−6.09*
**23**	4.11	2.11	6.77	.91	−8.39*

* p<.05

**Table 3:** Means, Standard Deviations and Alpha Coefficients for MSSC and PAI

**MSSC**	**α**	**M±Sd**	**PAI**	**α**	**M±Sd**
	**.87**			**.75**	
Vic.achievement	.72	6.26±1.17	Players	.88	2.41±1.82
Aesthetic	.76	6.21±1.07	Team	.85	5.82±1.51
Drama	.59	6.22±0.98	Coach	.72	2.69±1.45
Escape	.73	5.64±1.35	Commuinty	.73	4.20±1.91
Knowledge	.80	5.84±1.29	Sport type	.80	5.04±1.68
Physical skills	.61	6.18±0.99	City	.53	3.52±1.56
Social interaction	.73	5.39±1.52	Level of sport	.72	4.08±1.85

**Table 4:** Confirmatory Factor Analysis Results for MSSC and PAI

	**χ^2^**	**df**	**χ^2^/df**	**RMSEA**	**SRMR**	**CFI**	**GFI**	**R^2^**
**MSSC**	386.58	205	1.88	.067	.084	.91	.86	.11 – .75
**PAI**	335.25	163	2.05	.073	.086	.86	.85	.17 – .82

p<.01

**Table 5:** Measures of Overall Model fit for Canonical Correlation Analysis

	**Wilk’s**	**Chi-SQ**	**df**	**Canonical Correlation**	**p**
	
u1-v1	.528	120.41	49	.57	.000
u2-v2	.782	46.31	36	.34	.116
u3-v3	.888	22.37	25	.21	.614
u4-v4	.933	13.10	16	.18	.665
u5-v5	.967	6.31	9	.14	.708
u6-v6	.989	2.10	4	.09	.717
u7-v7	.998	.40	1	.04	.526

**Table 6:** Canonical Loadings and Canonical Cross Loadings for the Significant Canonical Function

**MSSC**	**Canonical Loadings**	**Cross Loadings**	**PAI**	**Canonical Loadings**	**Cross Loadings**
Vic. achievement	.366	.209	Players	.105	.060
Aesthetic	.779	.444	Team	.539	.307
Drama	.503	.287	Coach	.251	.143
Escape	.809	.461	Commuinty	.649	.370
Knowledge	.729	.416	Sport type	.809	.461
Physical skills	.614	.350	City	.490	.279
Social interaction	.393	.224	Level of sport	.429	.245

**Table 7:** Redundancy Index for MSSC and PAI

	**MSSC**	**PAI**
	
Variance explained by own variable	v1 = 0.387	u1 = 0.267
Variance explained by opposite variable	u1 = 0.126	v1 = 0.087
